# A DNA Aptameric Ligand of Human Transferrin Receptor Generated by Cell-SELEX

**DOI:** 10.3390/ijms22168923

**Published:** 2021-08-19

**Authors:** Nan Zhang, Tao Bing, Luyao Shen, Le Feng, Xiangjun Liu, Dihua Shangguan

**Affiliations:** 1 Beijing National Laboratory for Molecular Sciences, Key Laboratory of Analytical Chemistry for Living Biosystems, CAS Research/Education Center for Excellence in Molecular Sciences, Institute of Chemistry, Chinese Academy of Sciences, Beijing 100190, China; hszhang@iccas.ac.cn (N.Z.); bingtao@iccas.ac.cn (T.B.); shenluyao@iccas.ac.cn (L.S.); 18295497475@163.com (L.F.); xjliu@iccas.ac.cn (X.L.); 2 School of Chemical Sciences, University of Chinese Academy of Sciences, Beijing 100049, China; 3 School of Molecular Medicine, Hangzhou Institute for Advanced Study, University of Chinese Academy of Sciences, Hangzhou 310024, China

**Keywords:** aptamer, aptameric ligand, cell-SELEX, transferrin receptor, transcytosis

## Abstract

General cancer-targeted ligands that can deliver drugs to cells have been given considerable attention. In this paper, a high-affinity DNA aptamer (HG1) generally binding to human tumor cells was evolved by cell-SELEX, and was further optimized to have 35 deoxynucleotides (HG1-9). Aptamer HG1-9 could be taken up by live cells, and its target protein on a cell was identified to be human transferrin receptor (TfR). As a man-made ligand of TfR, aptamer HG1-9 was demonstrated to bind at the same site of human TfR as transferrin with comparable binding affinity, and was proved to cross the epithelium barrier through transferrin receptor-mediated transcytosis. These results suggest that aptamer HG1-9 holds potential as a promising ligand to develop general cancer-targeted diagnostics and therapeutics.

## 1. Introduction

A malignant tumor is still regarded as one of the most dreadful diseases this century, despite tremendous efforts being made to develop various cancer management strategies. Among current strategies, general cancer-targeted methods, hold great promise for cancer therapy and have been given considerable attention. Transferrin receptor I (TfR), also named CD71, is a type II transmembrane glycoprotein acting as a cell membrane receptor of human transferrin (hTf) [1,2]. TfR can continuously deliver extracellular iron-loaded hTf (holo-hTf) into cells, involved not only in iron homeostasis, but also in some physiological processes related to cell growth [3]. Besides being an essential iron transporter, TfR also plays critical roles in receptor-mediated endocytosis of multiple endogenous or extraneous ligands, such as ferritin and hereditary hemochromatosis factor (HFE) [4]. More and more literatures have documented that TfR is up-regulated in numerous kinds of tumors and its expression is associated with the malignancy of the tumor, such as metastasis and drug resistance [5]. Due to its ubiquitous expression in tumor cells and highly effective endocytosis, TfR becomes one of the most well-characterized cargo proteins and has been widely used as a universal tool to study targeted delivery of therapeutic agents against diseases [6,7,8]. hTf or anti-TfR antibodies covalently conjugated with chemical molecules or nanoparticles are the most used ligands for the study of TfR-mediated endocytosis or drug delivery [7,9]. However, the application of these conjugations is challenging with current technology, such as the difficulty in large scale preparation of hTf or antibodies, the complexity and uncertainty of the ligand conjugation on proteins, large molecular sizes of hTf (78 kDa) and antibodies (~150 kDa), and the sensitivities of the proteins to circumstance [8]. Thus, novel and simple ligands against TfR are needed.

Aptamers are single-stranded oligonucleotides which are generated by the Systematic Evolution of Ligand by EXponetial Enrichment (SELEX) technique [10]. Aptamers can recognize their targets with high affinity and selectivity in the way similar to antibodies. Recently, many aptamers and aptamer-drug conjugates have been developed and tested for clinical drug delivery due to their advantages, such as the relatively small and stable structures for modification in denatured conditions, high affinity, species specificity, and low immunogenicity [11], To date, some aptamers against TfR have been reported. Neufeld and coworkers reported two aptamers specifically against mouse TfR, which were used to deliver a lysosomal enzyme through receptor-mediated endocytosis [12]; however, these aptamers lacked affinity to human TfR. Levy and coworkers reported a 2′-F modified RNA aptamer that could recognize human TfR [13] and later reported another RNA aptamer could recognize the apical domain on human TfR [14]. These aptamers were applied for siRNA delivery and inhibition of the New World hemorrhagic fever mammarenavirus entry. Compared to RNA, DNA aptamer is more stable, lower cost, and easier to modify. Tan and coworkers reported a DNA aptamer against CD71 with 56 nucleotides (17.4 kDa) and an equilibrium dissociation constant (*K*_d_) of approximately 50 nM to CD71 and 82.5 nM to its target cell, this aptamer has been tested for unconjugated drug delivery [15,16]. However, more DNA aptamers against human TfR with high affinity, good specificity, and well-defined structure are still needed for the TfR-mediated drug delivery and TfR-related biomedical research.

Herein, we reported a DNA aptamer HG1-9, generated by cell-SELEX using drug-resistant colon cancer cell line HCT-8T as the target. The optimization and characterization of aptamer have been well conducted. The target protein of HG1-9 was identified to be human TfR by Stable Isotope Labeling by Amino acids in Cell culture (SILAC) based proteomic assay. To evaluate the potential of aptamer HG1-9 as an effective drug-carrier, the comparison of natural ligand hTf and aptamer HG1-9 in binding affinity and transcytosis efficiencies across epithelium barrier has also been investigated.

## 2. Results and Discussion

### 2.1. Selection of Aptamers against Malignant Tumor Cells

**Figure 1:**
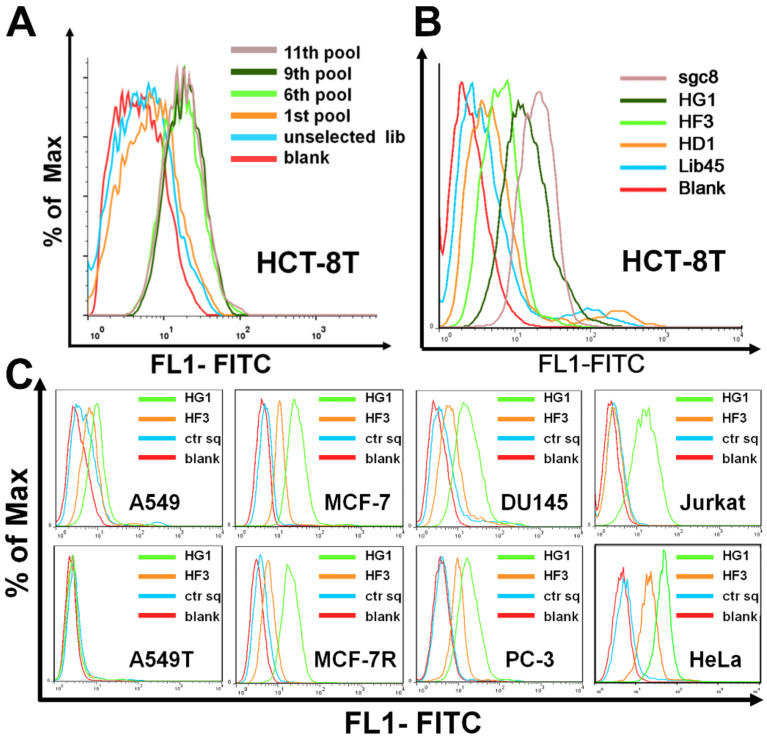
Monitoring the enrichment of cell-SELEX process. (**A**) Histogram of fluorescence intensity of enriched ssDNA pool on HCT-8T cells by flow cytometry, unselected DNA library was used as the negative control. (**B**) Histogram of fluorescence intensity of DNA sequences HG1, HD1, and HF3 on HCT-8T cells by flow cytometry, Lib45 was used as the negative control and PTK7-targeted aptamer sgc8 was the positive control. (**C**) Flow cytometry analysis of HG1 and HF3 binding to 8 different cell lines (A549, A549T, MCF-7, MCF-7R, DU145, PC-3, Jurkat, and HeLa cells). The ctr sq was as negative control.

Our original purpose was to generate aptamers that specifically bind drug-resistant cells. And for this purpose, a drug-resistant malignant tumor-taxol-resistant colorectal adenocarcinoma cell line (HCT-8T) was chosen as the target cell for aptamer selection, and its wild type HCT-8 cell line was used as the negative control cell. The selection process was performed as protocol previously reported [17]. As shown in Figure 1A, the sixth round selected pool presented strong fluorescence intensity on HCT-8T cells, suggesting a significant enrichment of ssDNA sequences bound to target cells; while the fluorescence intensity slightly increased from the seventh round pool to the 11th round pool. However, the evolved pools were also found to bind HCT-8 cells, suggesting the probable general cell-binding ability of the enriched pool (Figure S1, see Supplementary Materials). To prove this, eight tumor cell lines were further tested for binding with the 11th round pool. Interestingly, the results showed that the evolved pool, besides being bound to the target cell (HCT-8T), could also bind to another eight cell lines (including three solid tumor cell lines and their drug-resistant subtypes, a leukemia tumor cell line and an immortalized human embryo kidney cell line), indicating its general binding ability to tumor cells (Figure S2). Thus, the enriched 11th round pool was cloned. Then there were 50 clones chosen for sequencing (Figure S3). Among these 50 sequences, most of them were shortened and some were lengthened when compared with the original DNA library. This phenomenon has also been reported by other groups, and the length change of enriched sequences may happen during the PCR process [18]. After merging the repeated sequences, 19 sequences were analyzed and aligned by Clustal Omega program, three of the most abundant DNA sequences, HG1 (repeated 13 times), HD1 (repeated nine times) and HF3 (repeated eight times) were chosen as the representative aptamer candidates. The binding ability of HG1, HD1, and HF3 to target cells were examined by flow cytometry. As shown in Figure 1B, compared to controls, all the candidates could bind HCT-8T cells, while HG1 showed the strongest fluorescence. Moreover, cell-binding assay showed that HG1 could bind most of the tested human cell lines (A549, MCF-7, MCF-7R, DU145, PC-3, Jurkat, and HeLa cells) except A549T cells (taxol-resistant subline of A549 cell), suggesting the general binding ability of HG1 to tumor cells (Figure 1C). Since HG1 has the potential as a promising probe for generally recognizing tumor cells, further investigation of HG1 possesses its necessity.

### 2.2. Characterization and Optimization of Selected Aptamers

**Figure 2:**
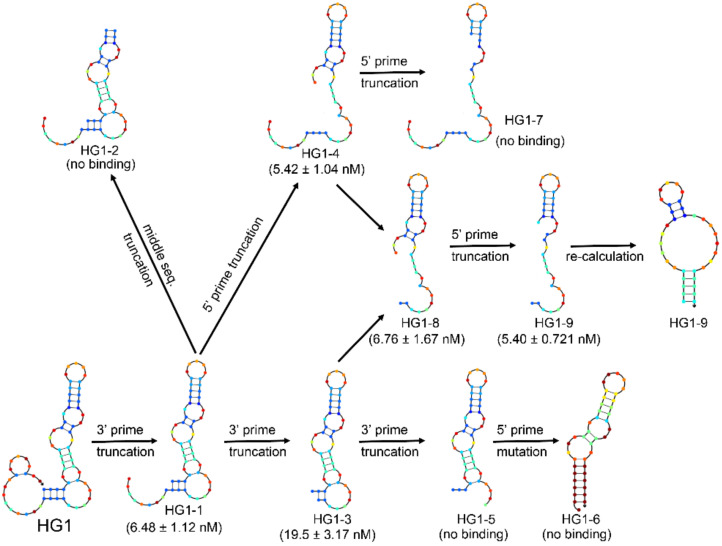
Truncation of HG1. The secondary structure of HG1 was predicted by the Nupack program and *K*_d_ of each sequence was given in parentheses.

In order to facilitate further application, HG1 was carefully optimized and characterized. As shown in Figure 2, the secondary structure of HG1 is composed of a complicated stem-loop structure with several bulges, predicted by Nupack [19]. In order to optimize this aptamer for more convenient use, we adopted an iterative truncation and mutation strategy. The sequence of HG1 was truncated respectively from the 5′ end, 3′ end, and middle sites according to the predicted secondary structure, and their binding abilities to HCT-8T cells were measured. The aptamer binding ability was not affected after truncating up to 15 deoxynucleotides from the 5′ end and 20 deoxynucleotides from the 3′ end of aptamer HG1; while the aptamer lost its binding ability after truncating several deoxynucleotides located at middle sites of HG1. Through this iterative truncation and mutation, an optimized aptamer, HG1-9, was obtained. Its sequence length was minimized from 70 nucleotides to 35 nucleotides, and its *K*_d_ decreased from 6.48 ± 1.12 to 5.40 ± 0.721 nM.

**Figure 3:**
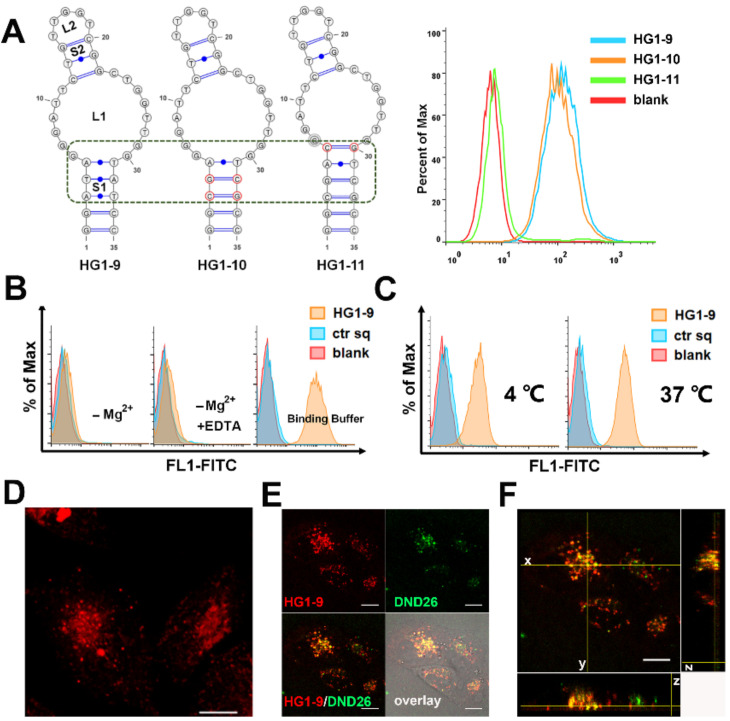
Characterization and optimization of HG1-9. (**A**) The predicted secondary structures of HG1-9, and its mutated sequences HG1-10 and HG1-11; and flow cytometry analysis of their binding to Jurkat cells. (**B**,**C**) The binding conditions that affect HG1-9 binding to Jurkat cells. HG1-9 incubated with cells at 4 °C in different buffers: PBS with 5 mM MgCl_2_, PBS without MgCl_2_and PBS with 5 mM EDTA (**B**). HG1-9 incubated with cells at temperatures of 4 and 37 °C in the binding buffer (**C**). Blank represents Jurkat cells without any treatment, ctr sq was the negative control. (**D**) 2D projection of the maximum intensity from axial (Z) dimension of fluorescence of HG1-9 (10 nM, labeled by TAMRA) within HeLa cells after incubation for 4 h at 37 °C. (**E**) Confocal imaging of HeLa cells co-stained by HG1-9 (labeled by TAMRA) and green Lysotraker (DND-26) at 37 °C for 30 min. (**F**) 3D images of HeLa cells in (**E**), the images respectively represent x–y plane, x–z plane and y–z plane. The scale is 10 μm.

HG1-9 was predicted to fold a secondary structure with two condensed stem-loops (S1, L1, S2, and L2, predicted by Nupack). In this structure, the stem S1 plays a role in maintaining the binding structure since mutation in stem S1 only slightly affected its binding ability, while mutation in L1 caused the loss of aptamer binding suggesting its involvement in binding domain (Figure 3A). This result suggests that conjugating drugs or reporters at the ends of aptamer HG1-9 or on the stem S1 would not significantly affect its binding ability. Since aptamers were selected in a binding buffer (containing 5 mM MgCl_2_) at 4 °C, the influences of divalent ions and temperature on the binding ability of HG1-9 was also investigated. As shown in Figure 3B, HG1-9 lost most of its binding ability in buffer without MgCl_2_ or in buffer with EDTA, suggesting that magnesium ion was essential for the target recognition of HG1-9. Moreover, the fluorescence intensity of cells incubated with HG1-9 in binding buffer at 37 °C for 30 min was stronger than that at 4 °C, while no significant fluorescence change of cells incubated with control sequence was observed, suggesting that a rising incubation temperature may facilitate internalization of HG1-9 into target cells (Figure 3C). To verify this, a 2D projection of the maximum intensity from the axial (Z) dimension (stacks of 2D images that show one image at a time) was presented [20].The fluorescence of aptamer HG1-9 was shown dispersedly in cells instead of the periphery of cells, suggesting that HG1-9 was taken up by cells (Figure 3D). To further investigate whether aptamer HG1-9 could be delivered into cells, HG1-9 and lysosome probe (DND-26) were co-stained HeLa cells at 37 °C, and 3D confocal imaging was conducted. Seen in Figure 3E,F, in addition to plane x–y, the fluorescence of HG1-9 and DND-26 could seldom overlay in either plane x–z and y–z, indicating HG1-9 could be easily taken up by cells but hardly entered into lysosomes.

### 2.3. Identification of Target Protein of Aptamer HG1-9

**Figure 4:**
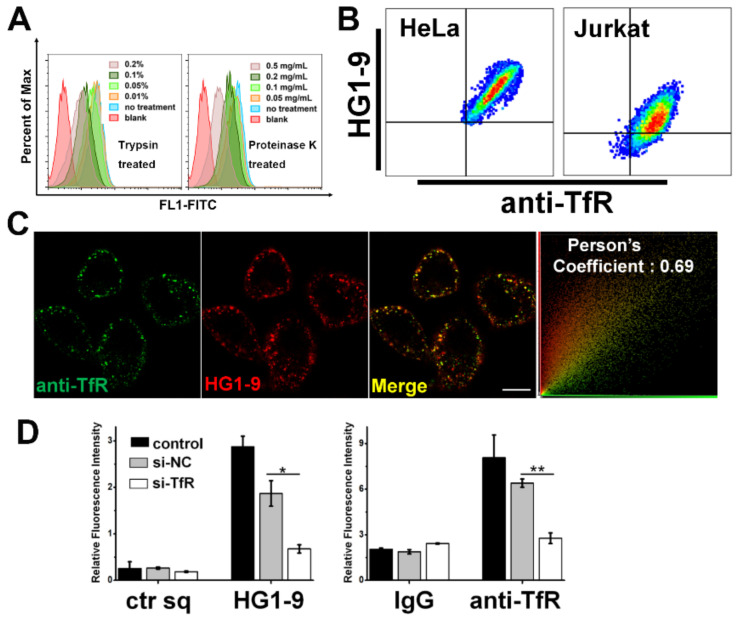
Identification of transferrin receptor as molecular target of aptamer HG1-9. (**A**) Flow cytometry analysis of HG1-9 binding to Jurkat cells pretreated by different concentrations of trypsin or proteinase K. (**B**) Flow cytometry analysis of HeLa and Jurkat cells dual-stained by aptamer HG1-9 (labeled by Cy5) and antibody anti-TfR (labeled by PE). The cross-quadrant gate in each bivariate histogram was respectively set according to negative control in different cell lines. (**C**) Confocal imaging of HeLa cells dual-stained by aptamer HG1-9 (labeled by FITC, 200 nM) and anti-TfR antibody (labeled by PE-second antibody, dilution of 1:20), the scale is 10 μm. (**D**) Relative median fluorescence intensities of aptamer HG1-9 and anti-TfR antibody binding to HeLa cells after siRNA knockdown. Relative median fluorescence intensities were acquired according to formula of F = (F1 − F0)/F0. Blank bars represent cells without any treatment, grey bars represent cells treated by si-NC sequence, and white bars represent cells treated by si-TfR. The ctr sq and IgG were as the negative control, *t*-test, *n* = 3, * *p* < 0.05; ** *p* < 0.01.

The general binding of HG1-9 to various tumor cells suggests that the molecular target of HG1-9 generally expressed on tumor cells, which might be a potential tumor biomarker. Thus, the identification of the molecular target on cell membrane was performed. As far as we know, most of previously reported aptamers, evolved by cell-SELEX, were found to bind the extracellular membrane proteins with few exceptions [21,22]. To preliminarily determine whether the molecular target of aptamer HG1-9 is a protein, the binding of aptamer HG1-9 to tumor cells treated by proteinases was investigated. After being digested by Trypsin or Proteinase K, the fluorescence intensity of HG1-9 on cells gradually decreased with the increase of proteinase concentration, suggesting that the molecular target of HG1-9 is a protein (Figure 4A).

Thus, to unbiasedly identify the target protein of aptamer HG1-9, a universal SILAC-based quantitative proteomic assay was employed [23]. After separately cultured in light (containing ^14^N, ^12^C-labeled lysine, and arginine) and heavy (containing ^15^N, ^13^C-labeled lysine, and arginine) media, approximately 10^8^ light- and heavy-tumor cells were harvested and respectively incubated with 5′-biotinylated aptamer HG1-9 and control sequence. After incubation and formaldehyde-mediated cross-linking, the cells were lysed; and HG1-9-protein complex was isolated, resolved, trypsin-digested and analyzed by LC-MS/MS. There were 24 protein candidates identified by MS. Among these identified proteins, Human Transferrin Receptor I; (TfR, or CD71) is the only protein with a protein abundance ratio (HG1-9/Lib45) higher than 20, while the protein abundance ratios of other 22 proteins are smaller than 2, and single stranded DNA binding protein 1 (SSBP1) is 6.5 ± 2.8 (Seen in Figure S4 and Table S2). SSBP1 can be excluded as the specific protein target of aptamer HG1-9 because it is not a membrane protein and can nonspecifically bind all ssDNA, since this protein was extracted in our previous work. Thus, Transferrin Receptor I; was as the most likely molecular target for further verification.

To validate that TfR is the protein target of aptamer HG1-9, anti-TfR antibody (labeled by phycoerythrin (PE)-conjugated second antibody) was used to co-stain cells with Cy5-labeled aptamer HG1-9. The flow cytometry analysis showed that both antibody and HG1-9 bound to TfR highly expressed cells (HeLa, Jurkat, SK-HEP-1, MCF-7, and A2780T) without competition, and the fluorescence intensities of anti-TfR and HG1-9 were positively correlated. In contrary, neither anti-TfR nor HG1-9 bound A549T cells (Figure 4B and Figure S6). Confocal imaging showed that the fluorescence of anti-TfR and HG1-9 overlaid well on the surface of HeLa cells, and the Pearson’s correlation coefficient (PCC) was calculated to be 0.69 (Figure 4C). And this fluorescence overlap could also be observed in Jurkat cells (Figure S5). Furthermore, the siRNA knockdown assay showed that the cellular binding of anti-TfR antibody and aptamer HG1-9 decreased sharply after treating HeLa cells with siRNA of TfR (si-TfR), suggesting that the protein target of HG1-9 was TfR (Figure 4D). Thus, all the results above prove conclusively that the target of HG1-9 is Human Transferrin Receptor I.

### 2.4. Comparison of Natural Ligand hTf and Aptamer HG1-9

**Figure 5:**
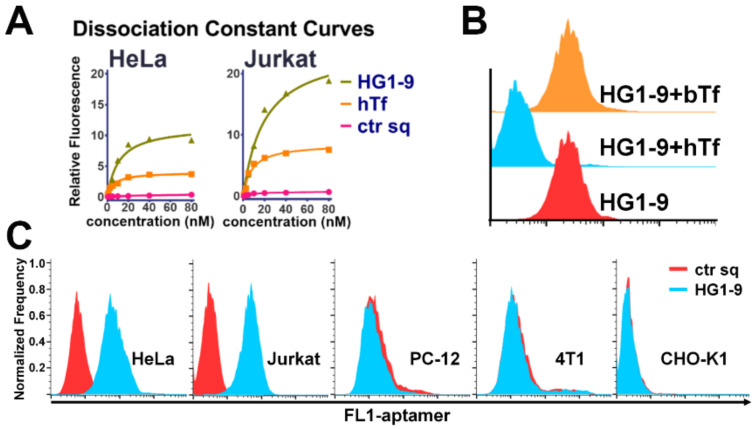
Comparison of hTf and aptamer HG1-9. (**A**) The dissociation constant curves of HG1-9 (labeled by FITC) and hTf (labeled by FITC) to Jurkat and HeLa cells, ctr sq was as the negative control. (**B**) Binding ability of HG1-9 (labeled by FITC, 200 nM) to HeLa cells under the competition of bovine Transferrin (bTf, 500 nM) or human Transferrin (hTf, 500 nM). (**C**) The binding of HG1-9 to human cancer cell lines (HeLa and Jurkat), and other cell lines (PC-12 (Rat adrenal pheochromocytoma), 4T1 (Mouse mammary carcinoma), and CHO-K1 (a subclone of Chinese hamster ovary cell)).

The natural ligand of TfR, hTf binds its receptor with high affinity, and has been widely applied as a drug-carrier [24]. Thus, the binding ability of HG1-9 and hTf to tumor cells were compared. The apparent *K*_d_s of fluorophore labeled apo-hTf (without iron-loaded) to Jurkat and HeLa cell were measured to be 5.98 ± 0.9 and 8.1 ± 1.8 nM (consistent with the previously reported results (<15 nM) [25]) (Figure 5A). The *K*_d_s of HG1-9 to Jurkat and HeLa cells were calculated to be 11.0 ± 2.9 and 19.8 ± 4.9 nM. These results suggest that HG1-9 possesses a considerable binding affinity to TfR on cell surface. Furthermore, the competition experiment showed that the addition of unlabeled holo-hTf (iron-loaded, 2.5-fold concentration of HG1-9) caused HG1-9 lost its binding ability to HeLa cells, but not affect the binding of anti-TfR antibody and another aptamer sgc8 (targeting PTK7) [10], suggesting that HG1-9 binds the same site on TfR with hTf (Figure 5B and Figure S7). What is more, bovine transferrin (bTf) was found not to affect the binding of HG1-9 at all. The further cell-binding assay showed that HG1-9 generally bound the tested human cell lines; but, did not exhibit notable binding ability to any non-human cell lines (PC-12, 4T1 and CHO-K1) those highly expressed TfR of their own species [26,27,28], suggesting good species specificity of aptamer HG1-9 (Figure 5C). This set of results proves conclusively that HG1-9 could bind human TfR on cell surface with good species specificity; HG1-9 and hTf recognize the same sites on TfR with comparable affinity.

### 2.5. The Transcytosis of Aptamer HG1-9 across Epithelium Barrier

**Figure 6:**
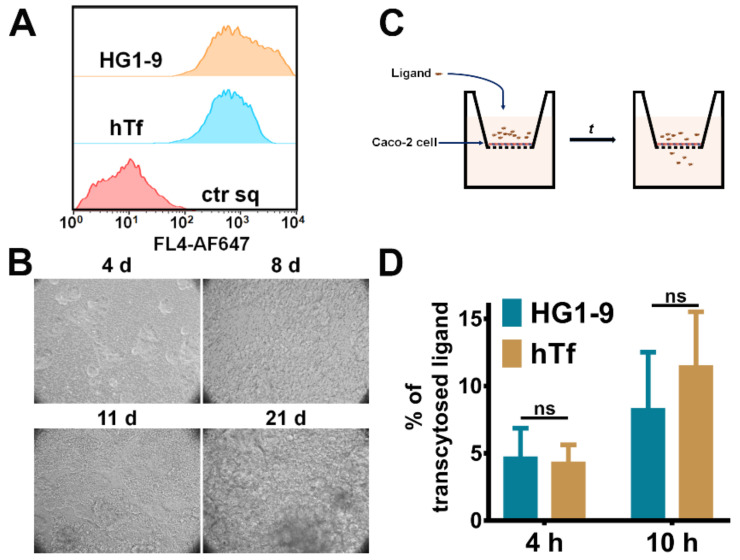
The transcytosis of aptamer HG1-9 across epithelium barrier. (**A**) Flow cytometry analysis of aptamer HG1-9 and hTf binding to Caco-2 cells at 4 °C. ctr sq was the negative control. All of the DNA sequences and proteins were labeled by AF647. (**B**) The monolayer of Caco-2 cells on Transwell plate at different cultured days. (**C**) The schematic diagram of transcytosis of HG1-9 and hTf in Caco-2 cells. (**D**) Histogram of transcytosis efficiencies of HG1-9 and hTf in Caco-2 cells after incubation for 4 and 10 h. All data were presented as mean ± SE, *n* = 4, *t*-test, ns, not significant.

The results above suggest that aptamer HG1-9 could bind to TfR at cell surface and be taken up into cells (Figure 3E,F). In order to figure out whether this man-made ligand of TfR could also be used as a carrier for drug delivery like hTf, the transcytosis efficiency of HG1-9 across epithelium barrier was evaluated. Caco-2 cells are enterocyte-like cells and often used as the intestinal epithelial cells to investigate the intracellular processing of internalized Tf [29]. Thus, an epithelium model was established by growing Caco-2 cell monolayer in a transwell culture plate. Firstly, the binding ability of hTf and HG1-9 to Caco-2 cells were verified by flow cytometry (Figure 6A). Then, Caco-2 cells were seeded on a polycarbonate film with 0.4-μm pores and cultured for 21 days to form a tight monolayer film (Figure 6B). Since Caco-2 cells are dipolar cells (i.e., the apical and basolateral sides of cells are different), fluorophore labeled ligands were added into the medium from the apical side, after incubation the fluorescence of the medium from the basolateral side was measured (Figure 6C). As shown in Figure 6D, the fluorophore labeled HG1-9 or hTf was respectively added into the upper chamber and incubated for 4 and 10 h, the concentrations of the ligands in the lower chamber were measured. There is no significant difference between the transcytosis efficiencies of the two ligands, suggesting that both HG1-9 and hTf could be transported from the upper chamber to the lower chamber and aptamer HG1-9 could overcome the epithelial barrier.

## 3. Materials and Methods

### 3.1. Materials

All of the DNA sequences (shown in Table S1) and siRNA sequences were synthesized and purified by Sangon Biotech Co., Ltd. (Shanghai, China). All fluorescence labeled sequences were labeled at the 5′-end. The purchased DNA freeze-dried powders were dissolved in Phosphate Buffer Saline (PBS), and the concentration of solutions was measured by an absorbance of 260 nm. Unless otherwise stated, the dissolved DNA solutions were heated at 95 °C for 5 min, cooled on ice for 10 min, and annealed at room temperature for at least 30 min. The annealed DNA solutions were stored at −20 °C. The purchased RNA freeze-dried powders were dissolved in DEPC-treated water and stored at −80 °C.

Anti-Transferrin Receptor antibody (ab108985, clone EPR4012) and rabbit IgG isotype control (ab172730, clone EPR25A) were purchased from Abcam, Inc. (Cambridge, UK). Iron-loaded human transferrin (holo-hTf) (T4132) was purchased from Sigma-Aldrich (St. Louis, MO, USA). Alexa Fluoro 647 (AF647) and Fluorescein (FITC) labeled hTf was purchased from Jackson ImmunoResearch Laboratories, Inc (Carlsbad, CA, USA). Lipofectamin^®^ RNAiMAX Reagent was purchased from ThermoFisher Scientific (Carlsbad, CA, USA). Green Lysotracker probe (DND-26) was purchased from Beyotime Biotechnology (Nantong, Jiangsu, China).

### 3.2. Cell Lines and Cell Culture

HCT-8 (human colon cancer), HCT-8T (taxol-resistant HCT-8 subline), A2780 (human ovarian cancer), and A2780T (taxol-resistant A2780 subline) cell lines were purchased from KeyGEN Biological Technology Co. Ltd. (Nanjing, Jiangsu, China). MCF-7 (human breast cancer), HEK293 (immortalized human embryonic kidney), and A549 (human non-small cell lung cancer) cell lines were purchased from Cell Resource Center of Shanghai Institute for Biological Sciences (Chinese Academy of Sciences, Shanghai, China). KB (human oral cancer), Jurkat E6-1 (Jurkat, human Acute T lymphoblastic leukemia), and Caco-2 (human colon cancer) cell lines were purchased from Cell Culture Center of Institute of Basic Medical Sciences, Chinese Academy of Medical Sciences (Beijing, China). SK-HEP-1 (human liver cancer), HeLa (human cervical cancer), PC-3 (human prostate cancer), and DU145 (human prostate cancer) cell lines were purchased from Typical Culture Preservation Commission Cell Bank, Chinese Academy of Sciences (Shanghai, China). MCF-7R (Doxorubicin-resistant MCF-7 subline), KB/VCR (Vincristine-resistant KB subline) cell line, and A549T (Taxol-resistant A549 subline) cell lines were purchased from Shanghai Aiyan Biological Technology Co. Ltd. (Shanghai, China). PC-12 (Rat adrenal pheochromocytoma) cell line, 4T1 (Mouse mammary carcinoma) cell line, and CHO-K1 (a subclone of Chinese hamster ovary cell) were gifts from Professor Jie Ma from the Chinese Academy of Medical Sciences. All of the cell lines were cultured in basic medium with 10% fetal bovine serum (FBS, Gibco) and 1% penicillin/streptomycin (Corning). A2780, MCF-7, HEK293, A549, and SK-HEP-1 cells were cultured in Dulbecco’s Modified Eagle Medium (DMEM, Gibco), CHO-K1 cells were cultured in Ham’s F12 Medium (Gibco), Caco-2 cells were cultured in MEM medium (Gibco), and the other cells were cultured in RPMI-1640 medium (Gibco). All of the cell lines were routinely cultured at 37 °C, in a humidified atmosphere with 5% CO_2_.

### 3.3. Procedures of Cell-SELEX

The initial library of selection was a single-stranded DNA oligonucleotide with two flanking regions of 20-nucleotide sequences for primer annealing and a central randomized region of 45-nucleotide sequences (5′-AAG GAG CAG CGT GGA GGA TA-N (45)-TTA GGG TGT GTC GTC GTG GT-3′). Two primers were used for PCR amplification: FAM-labeled forward primer (5′-FAM-AAG GAG CAG CGT GGA GGA TA-3′) and biotin-labeled reverse primer (5′-biotin-ACC ACG ACG ACA CAC CCT AA-3′).

The cell-SELEX procedures are performed generally according to previous reported protocol with minimal modification [17]. After scraped from cell dishes, target cells (HCT-8T) were incubated with annealed initial ssDNA pool (10 nmol) in binding buffer (PBS with 4.5 g/L glucose, 5 mM MgCl_2_, 1 mg/mL BSA, 0.1 mg/mL Herring sperm DNA) at 4 °C for 60 min. After incubation, the cells were washed with washing buffer (PBS with 4.5 g/L glucose and 5 mM MgCl_2_) to remove nonspecific binding. After elution, the bound ssDNAs were amplified by PCR, the amplified process is: 94 °C for 30 s, 60 °C for 30 s, and 72 °C for 30 s; after 8–12 cycles, the PCR products were elongated at 72 °C for 5 min. The PCR products were isolated by streptavidin-coated sepharose beads (GE Healthcare). After being desalted by NAP-5 column (GE Healthcare) and vacuum-dried, the FITC-labeled ssDNAs were used as ssDNA library for the next round of selection. After 6 rounds of selection, the ssDNA library was obviously enriched with bound sequences. The 11th selected ssDNAs were PCR-amplified with unlabeled primers. The unlabeled PCR products were cloned and sequenced. Cloning and sequencing were accomplished by Sangon Co., Ltd. (Beijing, China).

### 3.4. Flow Cytometry Analysis

The flow cytometry analysis was done to investigate the binding ability of aptamers or antibodies to cells under various kinds of treatment. Cells were dissociated with PBS containing 0.2% EDTA, and were harvested as monodispersed cell suspension. The cell suspension was centrifuged, washed and resuspended in binding buffer. For aptamer-staining, the fluorophore-labeled DNAs, after annealed, were incubated with the cell suspension for 30 min on ice in the dark. For antibody-staining, the cell suspension was pre-incubated with 1% BSA and incubated with monoclonal antibodies or isotype control for 30 min on ice in the dark. After incubation, the cell pellet was centrifuged, washed, and re-suspended. The cell samples were analyzed by a Becton Dickinson FACScalibur flow cytometer (Becton, Dickinson and Company, New York, NY, USA).

### 3.5. Confocal Microscopy Imaging

For fluorescence imaging, the cells were seeded in the glass cover bottomed-confocal dishes (NEST Biotechnology Co., Ltd., Wuxi, China). After being cultured for at least 24 h, the cells were washed and stained with dyes, aptamers, or antibodies on ice or at 37 °C. Confocal microscopy imaging was done with an OLYMPUS FV1000-IX81 confocal microscope (Olympus Corporation, Tokio, Japan) with 100×objective lens, and confocal images were processed by Olympus FV10-ASW 1.6 viewer software.

### 3.6. Proteinase Digestion

To determine the characteristics of aptamer’s binding target on extracellular surface of target cells primarily, the proteinase digestion protocol was done as below: Jurkat cells were washed twice with washing buffer, then digested by different concentrations of Trypsin or proteinase K at 37 °C for 5 min, respectively. The digestion was terminated by adding FBS. Jurkat cells without treated by proteinase as the positive control. The dispersed cells were washed twice with washing buffer, pre-cooled, and incubated with aptamer. Flow cytometry assay was applied as described in Section 3.4. Flow Cytometric Analysis.

### 3.7. SILAC-Based Proteomic Assay for Target Protein Identification

SILAC-based proteomic assay was used to identification of target protein of aptamer HG1-9. The detailed procedures was referred to the protocol previously reported [23]. After stable-isotope labeled, about 2 × 10^8^ Jurkat cells were collected to incubate with double-labeled aptamer HG1-9 with biotin on 5′-end and fluorescein on 3′-end. After incubation, 1% formaldehyde was used to induce cross-linking between target protein on cell surface and aptamers. Then the cells were lysed, and the supernatant was collected to further incubate with streptavidin-coated sepharose beads. The collected beads were washed, boiled and separated by SDS-PAGE. Then, the protein band on PAGE gel was excised, washed and trypsin-digested and analyzed by LC-MS and MS/MS experiments. The MS experiments were performed on an LTQ-Orbitrap Velos mass spectrometer (Thermo Fisher Scientific, San Jose, CA, USA). The original mass spectral data was searched using the MaxQuant search engine (version number: 1.5.5.1) in the UniProt database. The intensity ratios for heavy/light-labeled peptides of the candidate aptamer targets were further validated by manual analysis, where the intensity ratios were taken across the peaks found in the selected-ion chromatograms for precursor ions of the unique peptides derived from the candidate aptamer targets.

### 3.8. siRNA Transfection

To determine the target protein of aptamer, siRNA of human TfR (si-TfR, antisense: 5′-GCUGGUCAGUUCGUGAUUATT-3′; sense: 5′-UAAUCACGAACUGACCAGCTT-3′) and negative control sequence (si-NC, antisense, 5′-UUCUCCGAACGUGUCACGUTT-3′; sense, 5′-ACGUGACACGUUCGGAGAATT-3′) was used to knockdown the expression of TfR on cells, then the binding ability of these cells to aptamer HG1-9 was measured. Cells were seeded in a six-well plate and grown to 80% confluence. Then the culture medium was removed, and a new culture media with a transfection reagent of siRNA (si-TfR) or control sequence (si-NC) were added to the cells. The siRNA transfection reagent was prepared according to the Lipofectamine^®^ RNAiMAX Transfection Protocol. After transfection for 6 h, the cells were grown for another 48 h in new fresh media, the cells were harvested to measure the binding ability to the aptamer and the antibody through flow cytometry assay.

### 3.9. Investigation of the Transcytosis of hTf and HG1-9 by an Epithelium Model Established by Caco-2 Cells

Caco-2 cells were seeded on polycarbonate membrane of upper chamber of a transwell plate (Corning, transwell, 12 well, 0.4-μm pore) with a concentration of 1 × 10^5^ cells/well. After being cultured for 21 days, the epithelium model was established when the resistance became higher than 500 Ω/cm^2^. AF647 labeled hTf and HG1-9 (150 nM, 400 μL) were added into the upper chamber, and incubated with Caco-2 cells at 37 °C. Then, 100 μL of medium were taken out after incubation for 4 and 10 h, and the transcytosis proportions were calculated according to the calibration curves.

### 3.10. Statistical Analysis

The data needed to be quantified and were acquired from the experiments which were repeated at least two times and expressed as the mean ± S.E. Statistical analysis was performed by Origin 8.5.1. The *K*_d_ of the aptamer was determined by fitting the dependence of the fluorescence intensity of specific binding on the free concentrations of the aptamer to the equation of *Y* = *B*_max_ *X*/(*K*_d_ + *X*), using the Systat SigmaPlot 11 (San Jose, CA, USA) software. For target protein identification, the raw MS data were processed with the MaxQuant search engine (1.3.0.5), the analysis of these data was processed according to the protocol previously reported [23]. For significance analysis, *t*-test was used.

## 4. Conclusions

In this study, a DNA aptamer HG1 was generated by cell-SELEX. This aptamer was found to own the general binding ability to tumor cells and exhibited high binding affinity. The further optimized aptamer named HG1-9 had 35 deoxynucleotides. Magnesium ion was found to affect the aptamer’s binding ability. Temperature was found to affect the aptamer binding and cell uptake. Human Transferrin Receptor I; was identified to be the target molecule of HG1-9 through the SILAC-based quantitative proteomic analysis, which was further verified by aptamer-antibody dual-stained and siRNA knockdown experiments. Compared to hTf, HG1-9 was found to bind at the same sites on TfR with comparable affinity. Moreover, aptamer HG1-9 has been proved to cross the intestinal epithelium barrier through TfR-mediated transcytosis, indicating the potential as a carrier for drug delivery. In summary, the general recognition to tumor cells and effective transcytosis of aptamer HG1-9 make it a promising ligand to develop general cancer-targeted diagnostics and therapeutics.

## 5. Patents

D. Shangguan, N. Zhang, T. Bing, X. Liu, L. Shen, China Patent, ZL201610555387.6, 2016.

## Data Availability

Data sharing is not applicable to this article.

## Supplementary Materials

The following are available online at https://www.mdpi.com/article/10.3390/ijms22168923/s1.
