# Supplementary material for: A DNA Aptameric Ligand of Human Transferrin Receptor Generated by Cell-SELEX
Source: Int J Mol Sci. 2021 Aug 19;22(16):8923. doi: 10.3390/ijms22168923 (PMC8396340; doi:10.3390/ijms22168923)
Supplement: Supplementary file 1 [file ijms-22-08923-s001.zip › ijms-1322750-supplementary.pdf]

Supplementary Information

A DNA Aptameric Ligand of Human Transferrin Receptor Generated by Cell-SELEX

Nan Zhang [1,#], Tao Bing [1,2,#], Luyao Shen [1], Le Feng [1], Xiangjun Liu [1,2], and Dihua Shangguan [1,2,3,*]

[1] Beijing National Laboratory for Molecular Sciences, Key Laboratory of Analytical Chemistry for Living Biosystems, CAS Research/Education Center for Excellence in Molecular Sciences, Institute of Chemistry, Chinese Academy of Sciences, Beijing, 100190, China; hszhang@iccas.ac.cn (N.Z.); bingtao@iccas.ac.cn (T.B.); shenluyao@iccas.ac.cn (L. S.); xjliu@iccas.ac.cn (X. L.)

[2] School of Chemical Sciences, University of Chinese Academy of Sciences, Beijing, 100049, China

[3] School of Molecular Medicine, Hangzhou Institute for Advanced Study, University of Chinese Academy of Sciences, Hangzhou, 310024, China

* Correspondence: sgdh@iccas.ac.cn

Table S1. DNA sequences used in this work.

name	Sequences (5'-3')
HF	tttAAGCAGCGTGGAGGATATGCTTTCCGACCGTGTTCGTTTGTTATAACGCTGCTtt
ctr sq	AGAGCAGCGTGGAGGATAGTTGGGGTTTGGCAAGTATTG
sgc8	CTAACTGCTGCGCCGCCGGGAAAATACTGTACGGTTAGA
HF3	AAGGAGCAGCGTGGAGGATATGCTTTCCGACCGTGTTCGTTTGTTATAACGCTGCTTAGGG TGTGTCGTCGTGGT
HD1	AAGGAGCAGCGTGGAGGATAGGGTTGGGTTTGGTGTCTGGGTTGGCTGTTGGTGGCTTCC GGTTAGGGTGTGTCGTCGTGGT
HG1	AAGGAGCAGCGTGGAGGATAGGGATTCTGTTGGTCGGCTGGTTGGTATCCTTAGGGTGTGT CGTCGTGGT
HG1-1	AGGAGCAGCGTGGAGGATAGGGATTCTGTTGGTCGGCTGGTTGGTATCCTTAGGGTGTG
HG1-2	AGGAGCAGCGTGGAGGATGTTGGTCGGCTGGTTGGTATCCTTAGGGTGTG
HG1-3	AGGAGCAGCGTGGAGGATAGGGATTCTGTTGGTCGGCTGGTTGGTATCC
HG1-4	GTGGAGGATAGGGATTCTGTTGGTCGGCTGGTTGGTATCCTTAGGGTGTG
HG1-5	AGGAGCAGCGTGGAGGATAGGGATTCTGTTGGTCGGCTGGTTGGT
HG1-6	TAACCAGCGTGGAGGATAGGGATTCTGTTGGTCGGCTGGTT
HG1-7	TAGGGATTCTGTTGGTCGGCTGGTTGGTATCCTTAGGGTGTG
HG1-8	GTGGAGGATAGGGATTCTGTTGGTCGGCTGGTTGGTATCC
HG1-9	GGATAGGGATTCTGTTGGTCGGCTGGTTGGTATCC
HG1-10	GGCGAGGGATTCTGTTGGTCGGCTGGTTGGTCGCC
HG1-11	GGCGACGGATTCTGTTGGTCGGCTGGTTGGTCGCC
c-HG1-9	GGATACCAACCAGCCGACCAACAGAATCCCTATCC
c-ctr sq	CAATACTTGCCAAACCCCAACTATCCTCCACGCTGCTCT

Table S2. Results of identification of target proteins by SILAC-MS method. TfR was the only protein with abundance ratio (HG1-9 / ctr) greater than 20.

Refseq protein accession	*Name of gene*	*Description*	*Number of Specific peptides*	*Coverage rate of sequences [%]*	*PEP*	*Abundance ratio of protein (HG1-9 /ctr sq)*[b]
NP_003225	TFRC	Transferrin receptor protein 1	18	23.8	4.7E-59	>20
NP_071415	MCCC2	3- methyl CoA coenzyme A carboxylase β chain	25	52.9	0.0E+00	0.9±0.2
NP_064551	MCCC1	3- methyl CoA coenzyme A carboxylase α chain	22	33.9	0.0E+00	0.9±0.1
NP_000273	PCCA	propionyl-CoA carboxylase alpha subunit	39	52.7	0.0E+00	0.9±0.2
NP_942131	ACACA	acetyl-CoA carboxylase alpha	94	51.5	0.0E+00	0.9±0.2
NP_071504	PC	pyruvate carboxylase	61	67.3	0.0E+00	1.0±0.1
NP_000402	HLCS	holocarboxylase synthetase	11	15.8	6.7E-80	1.0±0.2
NP_000523	PCCB	propionyl-CoA carboxylase beta subunit	28	62.1	0.0E+00	0.9±0.2
NP_003312	TUFM	Tu translation elongation factor, mitochondrial	10	27.3	1.3E-76	0.8±0.2
NP_006784	PRDX3	peroxiredoxin 3	3	11.3	5.9E-12	0.9±0.0
NP_000013	ADA	adenosine deaminase	6	27.3	9.7E-49	0.8±0.1
NP_066964	XRCC5	X-ray repair cross complementing 5	12	21.3	4.2E-50	1.5±0.7
NP_006089	GNB2L1	guanine nucleotide binding protein, beta 2, related sequence 1	4	15.5	7.4E-12	0.9±0.3
NP_003134	SSBP1	single stranded DNA binding protein 1	8	58.1	1.4E-126	6.5±2.8
NP_004125	HSPA9	heat shock protein family A (Hsp70)	11	18.1	8.6E-86	0.9±0.3
NP_005909	MDH2	malate dehydrogenase 2	5	19.5	6.2E-11	1.0±0.1
NP_006073	TUBA1B	tubulin alpha 1b	11	27.7	4.7E-120	1.1±0.4
NP_002148	HSPE1	heat shock protein family E (Hsp10) member 1	4	29.4	4.4E-21	0.8±0.2
NP_002406	MIF	macrophage migration inhibitory factor	2	17.4	3.2E-14	1.4±0.3
NP_002147	HSPD1	heat shock protein family D (Hsp60) member 1	29	57.1	7.8E-298	0.8±0.2
NP_002159	IDH2	isocitrate dehydrogenase (NADP(+)) 2, mitochondrial	7	14.4	3.7E-20	0.9±0.3
NP_000468	ALB	albumin 69 kDa	5	13.9	3.2E-44	0.8±0.7
NP_006588	HSPA8	heat shock protein family A (Hsp70) member 8	14	29.1	1.5E-93	1.8±0.9
NP_859047	PRDX1	peroxiredoxin 1	5	22.1	4.0E-15	1.1±0.4

[a] PEP represents posteriori error estimation; [b] represents ratio of forward sample and backward sample, the data were repeated two times and expressed as means ± S.E.

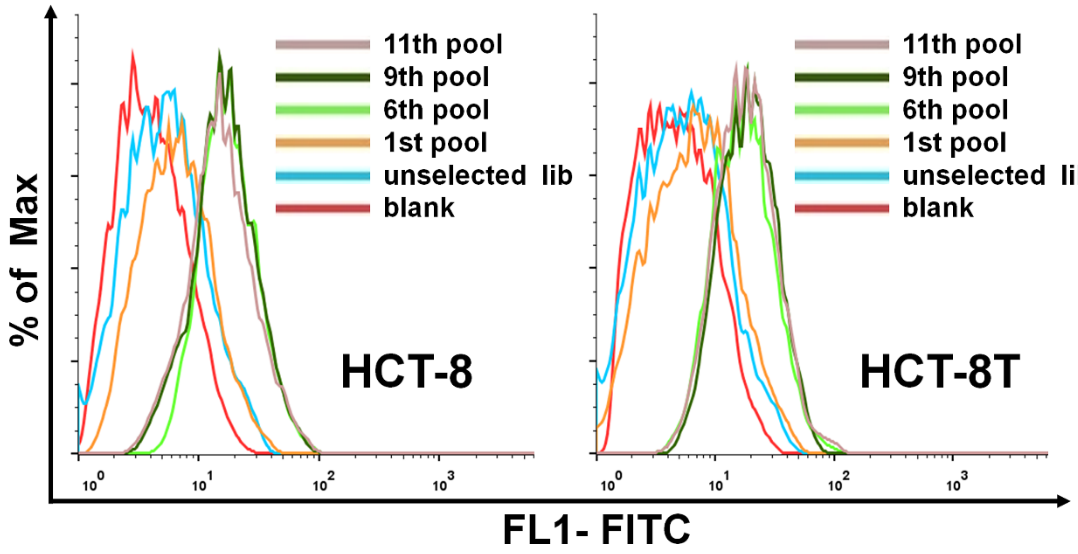

Figure S1. Monitoring enrichment of cell-SELEX process. Histogram of fluorescence intensity of selected ssDNA pool on HCT-8 (A) and HCT-8T (B) by flow cytometry. Unselected lib is as the negative control.

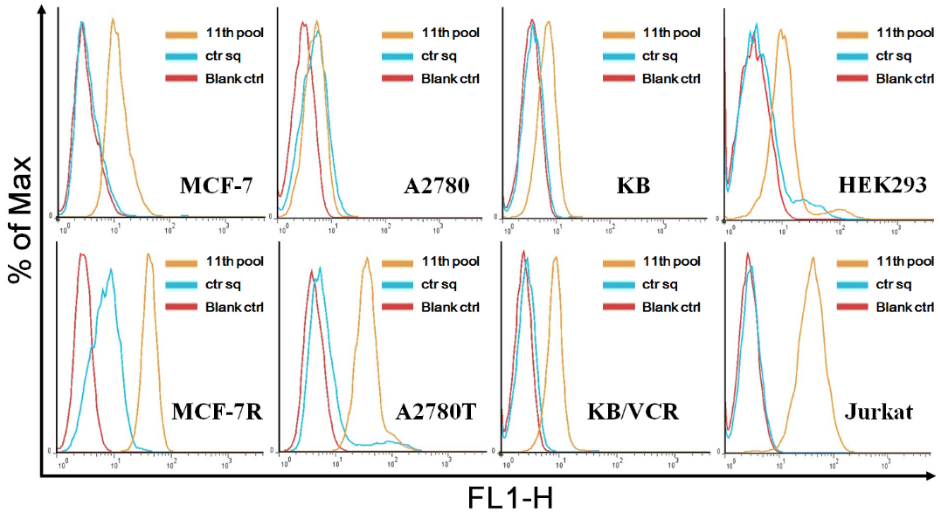

Figure S2. Flow cytometry analysis of the evolved 11th pool binding to different cell lines. The ctr sq was as negative control.

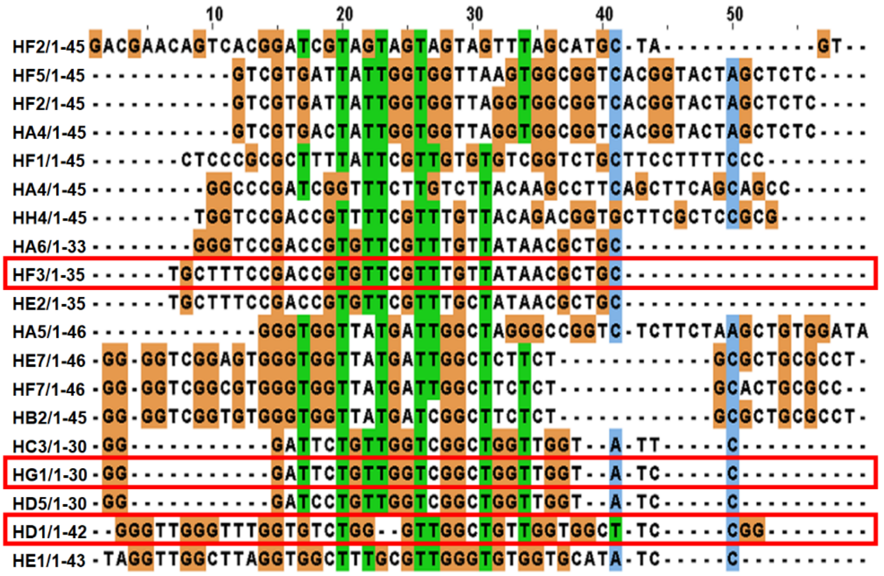

Figure S3. 19 cloned sequences (figure shows the random parts) were analyzed and aligned up by Clustal Omega program after merging the repeated sequences. The sequences in red boxes are HF3 (repeated 8 times), HG1 (repeated 13 times) and HD1 (repeated 9 times) are most abundant DNA sequences.

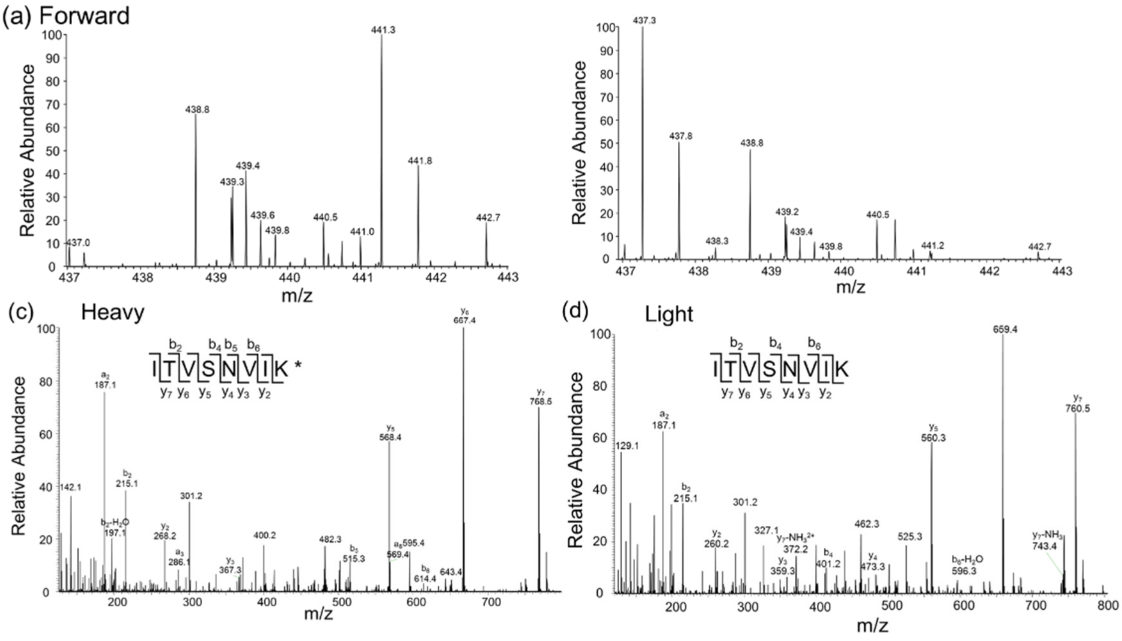

Figure S4. Representative ESI-MS and MS/MS of a tryptic peptide from human Transferrin Receptor. K* designates the heavy lysine. Shown in (a) and (b) are the ESI-MS for the heavy (m/z 441.3 for the monoisotopic peak of the $[M+2H]^{2+}$ ion) and the light (m/z 437.3 for the monoisotopic peak of the $[M+2H]^{2+}$ ion) lysine-containing peptide observed in forward and reverse SILAC experiments. Displayed in (c) and (d) are the MS/MS for the $[M+2H]^{2+}$ ions of the heavy- and light-lysine-bearing peptide.

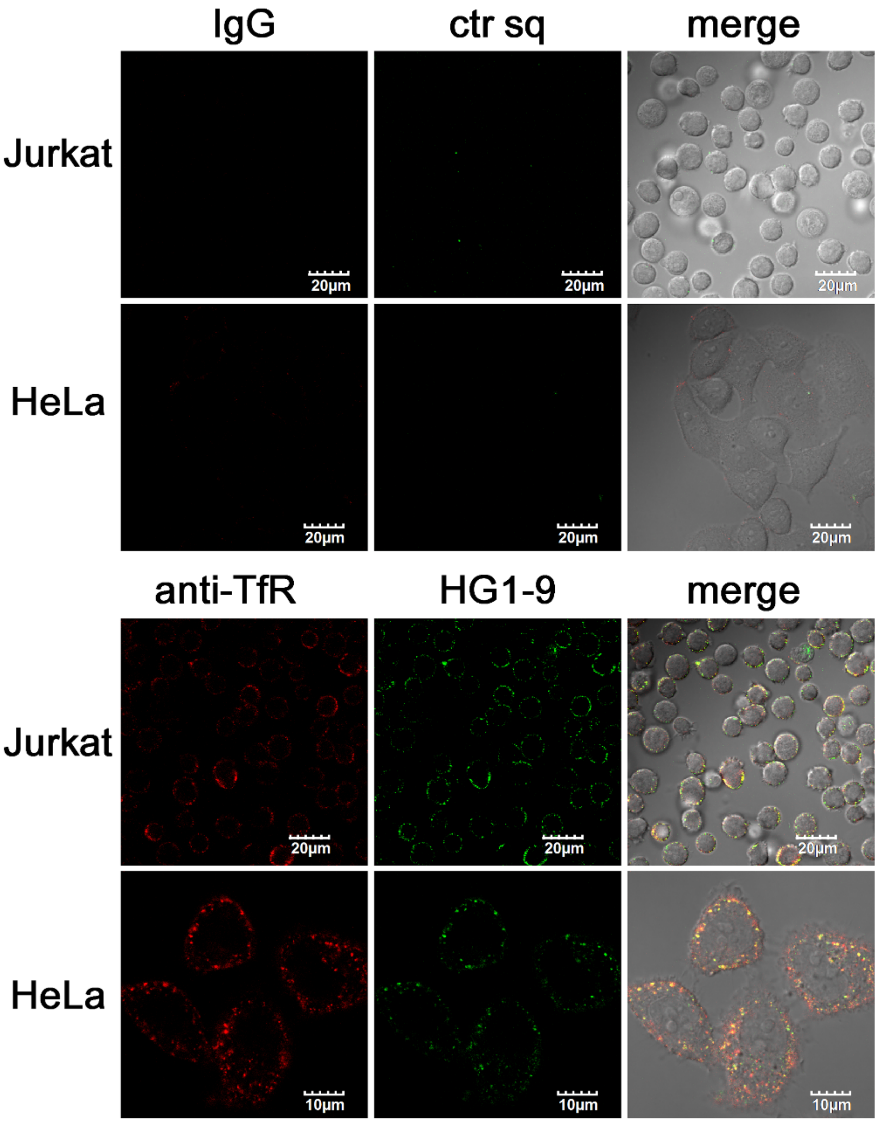

Figure S5. Confocal imaging of Jurkat and HeLa cells dual-stained by aptamer HG1-9 (labeled by FAM) and antibody anti-TfR (labeled by PE), and ctr sq (labeled by FAM) and iso-type control IgG (labeled by PE).

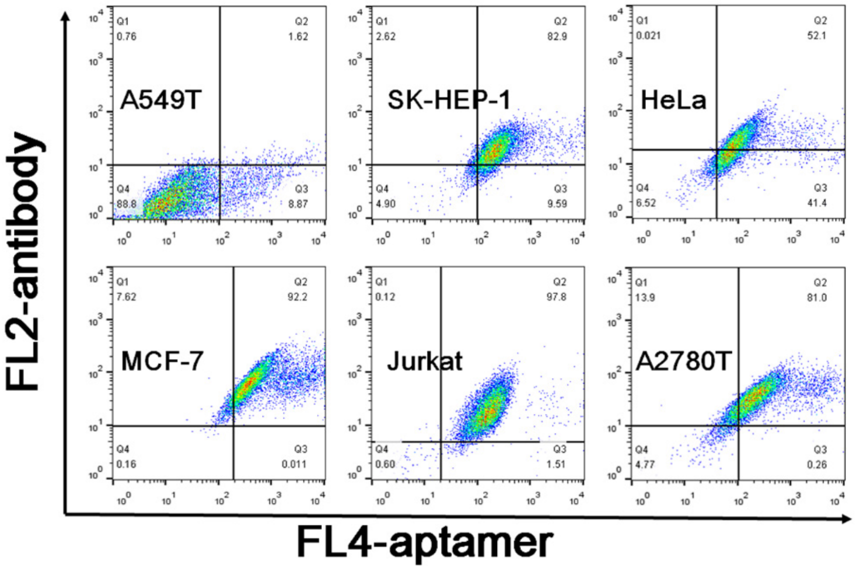

Figure S6. Flow cytometry analysis of different cell lines dual-stained by aptamer HG1-9 (labeled by Cy5) and antibody anti-TfR (labeled by PE). The cross-quadrant gate in each bivariate histogram was respectively set according to negative control in different cell lines.

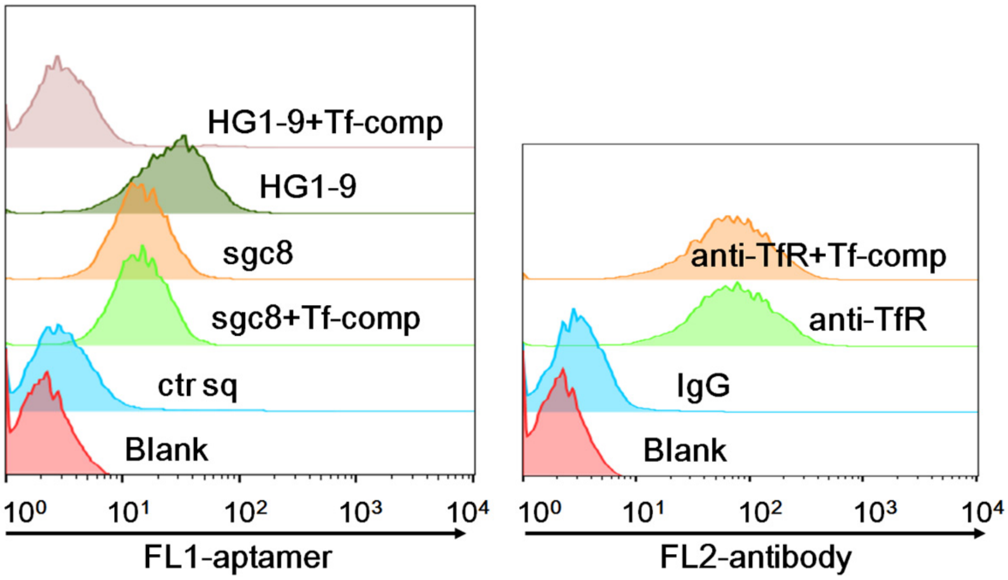

Figure S7. Flow cytometry analysis of aptamer and antibody binding to Jurkat cells with or without competition to holo-Tranferrin (Tf). Aptamer sgc8 was the negative control.
